# Part-List Cues Hinder Familiarity but Not Recollection in Item Recognition: Behavioral and Event-Related Potential Evidence

**DOI:** 10.3389/fpsyg.2020.561899

**Published:** 2020-10-02

**Authors:** Tuanli Liu, Min Xing, Xuejun Bai

**Affiliations:** ^1^School of Education Science, Xinyang Normal University, Xinyang, China; ^2^Key Research Base of Humanities and Social Sciences of the Ministry of Education, Academy of Psychology and Behavior, Tianjin Normal University, Tianjin, China; ^3^Faculty of Psychology, Tianjin Normal University, Tianjin, China

**Keywords:** recognition, recollection, familiarity, part-list cuing effect, FN400, late positive complex

## Abstract

Participants’ memory performance is normally poorer when a subset of previously learned items is provided as retrieval cues than none of the retrieval cues is provided. This phenomenon is called the part-list cuing effect, which has been discovered in numerous behavioral studies. However, there is currently no relevant behavioral or event-related potential (ERP) research to investigate whether the forgetting effect caused by part-list cues is more sensitive to recollection or to familiarity. By combining the part-list cuing paradigm with the Remember/Know procedure, we investigated this issue in the present ERP study. Behavioral data showed part-list cuing induced detrimental effect in two aspects: significantly lowered familiarity of the target items and decreased memory discrimination score (*P_r_* score) for “Know” but not for “Remember” items in the part-list cue condition than in the no-part-list cue condition. ERP data revealed that the FN400 old/new effects, which are associated with familiarity, were absent when providing part-list cues, whereas the late positive complex (LPC) old/new effects, which are associated with recollection, were observed comparably in both part-list cue and no-part-list cue conditions. Converging behavioral and ERP results suggested that part-list cues hindered familiarity-based retrieval but not recollection-based retrieval of item recognition. Theoretical implications of the findings for the part-list cuing effect are discussed.

## Introduction

Memory retrieval is influenced by multiple factors (Baddeley et al., 2014), among which is the adequacy of retrieval cues (Tulving and Pearlstone, 1966). However, retrieval cues are not always conductive to item recalling. When providing a portion of items from a previously studied list as retrieval cues and asking people to recall the remaining items, people often do more poorly on that list than do people asked to recall the items without the presence of such retrieval cues (Slamecka, 1968; Bäuml and Samenieh, 2012; Radvansky and Tamplin, 2013; Barber et al., 2015; Aslan and John, 2019; John and Aslan, 2020). This phenomenon is called the part-list cuing effect, which has been well documented by behavioral studies.

The part-list cuing effect is often presumed to result from inhibitory control mechanisms. The retrieval inhibition hypothesis regarded the part-list cuing effect as an aftereffect of the inhibitory executive-control processes that supposedly suppress the non-cue (target) items’ memory representation due to the presentation of part-list cues (Anderson et al., 1994; Bäuml and Aslan, 2004, 2006). That is, part-list cues lead to implicit retrieval of the cue items in the recall process, which in turn inhibits the overall strength of the non-cue items (Crescentini et al., 2010; Barber et al., 2015). Inhibition directly affects the representation strength of non-cue items; therefore, regardless of the type of retrieval cues used as probing words for retrieval and regardless of the type of output order provided by experimenter, the recalling of target items will be impaired or reduced. The inhibition account considers the harmful effects of part-list cues on memory retrieval to be persistent because it reflects a long-term changes in the activation level of the target items (Bäuml and Aslan, 2006; Mickes et al., 2013).

Another explanation for the part-list cuing effect is the strategy disruption hypothesis, which postulates that the presentation of part-list cues interferes with the memory strategy developed by subjects during the encoding phase, thus leading to a decline in memory scores (Basden and Basden, 1995; Reysen and Nairne, 2002). The less the memory strategy used during the retrieval phase resembles that of the encoding phase, the more the interference will be. The part-list cues reduce the similarity level of strategies, thus forcing the subjects to either develop a new strategy or exert more effort to recover their original strategies (Basden et al., 2002; Aslan and Bäuml, 2007). Therefore, according to this hypothesis, controlling output order means asking participants to use experimenter-manipulated retrieval strategies, which will cause a decline in memory performance regardless of whether the part-list cues are provided (Aslan and Bäuml, 2007). The strategy disruption hypothesis considers the effect of part-list cues on memory retrieval to be short-lived, that is, if part-list cues are removed, the cue-induced forgetting should be eliminated (Bäuml and Aslan, 2006).

Currently, such part-list cuing impairment has been demonstrated under a wide variety of manipulations, in semantic and episodic memory (Brown, 1968; Sloman et al., 1991), in vertical and false memory (Reysen and Nairne, 2002; Bäuml and Kuhbandner, 2003; Kimbal et al., 2008), in laboratory and real-world contexts (Pei and Tuttle, 1999; Bierstaker, 2003; Bovee et al., 2009), in high associative and low associative encoding situations (Bäuml and Aslan, 2006; Aslan and Bäuml, 2009; Muntean and Kimball, 2012; Lehmer and Bäuml, 2018; John and Aslan, 2020), in intra-list and extra-list cues conditions (Basden et al., 1977; Roediger et al., 1977; Peynircioğlu, 1989), in healthy and clinical subjects (Bäuml et al., 2002; Kissler and Bäuml, 2005; Christensen et al., 2006), and among different age groups (Marsh et al., 2004; Christensen et al., 2006; Andrés, 2009; Andrés and Howard, 2011; John and Aslan, 2018, 2020; Aslan and John, 2019).

However, an overwhelming majority of prior work focusing on part-list cuing effect mainly adopted free recall task or item-specific probe test as the measure of memory performance. Therefore, the theories proposed to explain the part-list cuing effect were mainly based on the results from free recall test or item-specific probe test. Only a few studies (Slamecka, 1975; Todres and Watkins, 1981; Oswald et al., 2006) have explored the part-list cuing effect in a classic yes/no recognition test, in which, however, the recollection and familiarity process cannot be behaviorally dissociated. According to a widely recognized dual-process recognition memory theory, recognition memory performance reflects two distinct memory processes: recollection and familiarity (Yonelinas and Jacoby, 2012; Pergola and Suchan, 2013; Tousignant et al., 2015; Bader and Mecklinger, 2017; Li et al., 2017; Bastin et al., 2019). Recollection is the retrieval of details associated with the previously experienced event; based on recollection, the spatial-temporal contextual information of the event and other information related to the event can be remembered and recalled. In contrast, familiarity is the feeling of having encountered the target event previously without the retrieval of additional contextual or associated information (Costanzo et al., 2013; Johnson et al., 2013; King et al., 2018). Currently, none of the abovementioned studies touches on the issue of whether the forgetting effect caused by part-list cues are more sensitive to recollection or familiarity process in recognition memory, given that free recall and item-specific probe recall are supposed to rely primarily on the recollection process.

We therefore sought to investigate how the part-list cues hinder recognition memory and which recognition process (recollection or familiarity) would be affected. For this purpose, the Remember/Know(R/K) procedure, a widely used paradigm for recognition memory to dissociate recollection with familiarity was adopted (Tulving, 1985; Evans and Wilding, 2012; Mickes et al., 2013; Voss and Paller, 2017; King et al., 2018; Ventura-Bort et al., 2020). In the standard R/K recognition task, each recognized target item was classified as R or K based on participants’ subjective memory experience. When participants can recognize the specific item and recollect details about their study experience with it, then an R judgment is given to that item (indicating an experience of episodic recollection); when participants have sufficient familiarity to provide adequate ground for a recognition judgment and the details about the study experience cannot be recalled, then a K judgment is given to that item (indicating a mere feeling of familiarity) (Rosenstreich and Goshen-Gottstein, 2015; Gao et al., 2019). For example, when it comes to remember, it means that when we retrace our memories back to last night, we can recall what we did last night and many related details of these things. When it comes to know, it means that we know things that we have experienced in the past, such as a phone number, but there is no specific memory about where they came from.

By employing the event-related potential (ERP) technique, researchers put forward representative neural correlates to indicate recollection and familiarity, respectively (Curran, 2000, 2004; Küper and Zimmer, 2018; Horne et al., 2020). It has been widely accepted that the FN400 (negative ERPs peaked at 300–500 ms post-stimulus onset) old-new effect at the frontal area reflected the familiarity process, that is, the old item elicited more positive ERP component than did the new item, whereas the late positive complex (LPC; positive ERPs peaked at 500–700 ms post-stimulus onset) old-new effect at the parietal area indexed the recollection, that is, the old item elicited larger LPC than did the new item (Curran, 2000, 2004; Rugg and Curran, 2007; Friedman et al., 2010; Stróżak et al., 2016b; Bader and Mecklinger, 2017; Li et al., 2017; Küper and Zimmer, 2018; Mecklinger and Bader, 2020). Although FN400 is considered to be similar in timing and morphology to N400, a perception/conceptual priming-related correlate (Voss and Paller, 2009; Meyer et al., 2010; Voss et al., 2010, 2012; Kutas and Federmeier, 2011; Dew and Cabeza, 2013; Hou et al., 2013; Pergola et al., 2014; Wang et al., 2015), most of the previous studies have shown many times that FN400 indicates the familiarity process in recognition memory (Wang et al., 2012; Kamp et al., 2016). Furthermore, it is widely believed that there is a functional dissociation between conceptual priming and familiarity, and they each depend on at least partial distinct neural regions (Johnson et al., 2013; Pergola and Suchan, 2013; King et al., 2018; Bastin et al., 2019; Barker and Warburton, 2020). Recent studies have suggested that these two components can be dissociated based on the distribution of topography (Bridger et al., 2012; Stróżak et al., 2016a; Bader and Mecklinger, 2017; Mecklinger and Bader, 2020). Therefore, in the present study, the two old/new effects of FN400 and LPC were adopted as the electrophysiological indicator of familiarity and recollection.

The part-list cuing effect is fundamentally a harmful effect of part-list cues on retrieval process. So the investigation of the retrieval process itself is a more direct way to figure out the mechanism of part-list cuing effect. However, as far as we know, no study to date has directly investigated the effect of part-list cues on recognition process, especially the effects of part-list cues on ERP measures during recognition of formerly studied material. Therefore, we combined the similar part-list cuing paradigm used by Oswald et al. (2006) with an R/K task identical to prior studies (Schaefer et al., 2011; Hou et al., 2014; Shaffer and Mcdermott, 2020) to investigate neurocognitive mechanism of part-list cuing effect. Specifically, which recognition processes (familiarity vs. recollection) could be affected by part-list cues should be demonstrated. Given that the memory performance decreased in previous studies using yes/no recognition task (Todres and Watkins, 1981; Oswald et al., 2006), we investigated the extent to which the behavioral memory accuracies and ERP amplitudes of FN400 and/or LPC effects would be decreased by part-list cues.

Previous studies have revealed the determinate effect of the output order and the duration of part-list cues on the cause of part-list cuing effect (Bäuml and Aslan, 2004; Aslan and Bäuml, 2007; Bäuml and Samenieh, 2012; John and Aslan, 2020). In the present study, the part-list cues were removed after 90-s presentation in the part-list cue condition, and the output order was controlled by the recognition task. Therefore, different predications can be derived from the two hypotheses of part-list cuing effect: at the behavioral level, significant lowered recognition performance according to the retrieval inhibition hypothesis versus no such significant decreased performance according to the strategy disruption hypothesis; at the electrophysiological level, old/new effect would change under the part-list cue condition compared to the no-part-list cue condition according to the retrieval inhibition hypothesis versus no such significant change according to the strategy disruption hypothesis.

## Materials and Methods

### Participants

Eighteen students participated in the experiment. Sample size was based on prior ERP studies related to Remember/Know recognition (Wang et al., 2016; Küper and Zimmer, 2018; Horne et al., 2020). The data from one participant had to be excluded due to excessive Electroencephalogram (EEG) artifacts. The mean age of the remaining 17 participants was 20.64 years (*SD* = 2.80, range 18–27, 7 males). All participants were right-handed and had normal or corrected-to-normal vision; none of them reported any personal or family history of psychiatric or neurological disorder. All participants signed an informed consent approved by the Human Research Ethics Committee of Xinyang Normal University and were paid for their participation.

### Stimuli

The materials consisted of 13 exemplars from each of the 54 semantic categories (*Animal, Math operation, Equipment-hiking, Folk art, Auto parts, Royalty member, Season, Jewelry, Food flavoring, Dog, Time unit, Sport, Furniture, Science, Non-relative relations, Weapon, Diseases, Non-alcoholic beverage, Flower, Emotion, Clothing, Insect, Tool Carpenter’s, Reading materials, City, Motion, Occupation, Natural earth formation, Fruit, Human body, Crime, Appliance-major, Building, Musical instrument, Cloth, Toy, Fish, Equipment-farm, Dance, Vehicle, Mythical being, Beverage-alcoholic, Herb, Tree, Shape, Building material, Country, Bird, Writing implement, Fuel type, Nut, Religious object, Vegetable, and Cosmetic*) that were obtained from the published norms (Yoon et al., 2004) in which 105 categories were identified for the subjects of Chinese adults. Exemplars for each category were ranked according to their strength of association with the category label. Each category’s exemplars were generally the 13 strongest associates to the category label according to the norms.

Six study-test blocks were constructed, each consisting of nine semantic categories. In each block, in the learning phase, 10 intermediate rank-ordered items (2–11 or 3–12) from each of the nine categories were chosen to be the study items. These items were presented in category-exemplar [e.g., 职业-演员(occupation-actor)] format. Four exemplars from each of the nine categories presented in the learning phase were chosen for use as part-list cues. The remaining 54 items served as old items during the recognition phase. The highest rank-ordered (1 or 1–2) and lowest rank-ordered (12–13 or 13) three items from each of the nine categories were chosen as new items during the recognition phase. Three blocks were assigned to the part-list cue condition, and the other three blocks were assigned to the no-part-list cue condition. Blocks were counterbalanced across cue conditions.

### Procedure

The experiment was programmed by Eprime1.1 software and presented on a 21-inch CRT screen (resolution ratio: 1024 × 768, refresh rate: 85 Hz).

During ERP recordings, stimuli were presented on a computer screen approximately 1 m away from the participants; stimuli subtended a visual angle of approximately a maximum horizontal visual angle of 4.6° and a maximum vertical visual angle of 0.8°. Subjects were seated in a comfortable armchair in a sound-dampened, homothermal room to reduce systematic error and accidental error during data collection. In order to minimize ERP artifacts, participants were required to blink as infrequently as possible, to minimize body and eye movements, and to keep their feet flat on the floor during performing the designated task.

**Figure 1:**
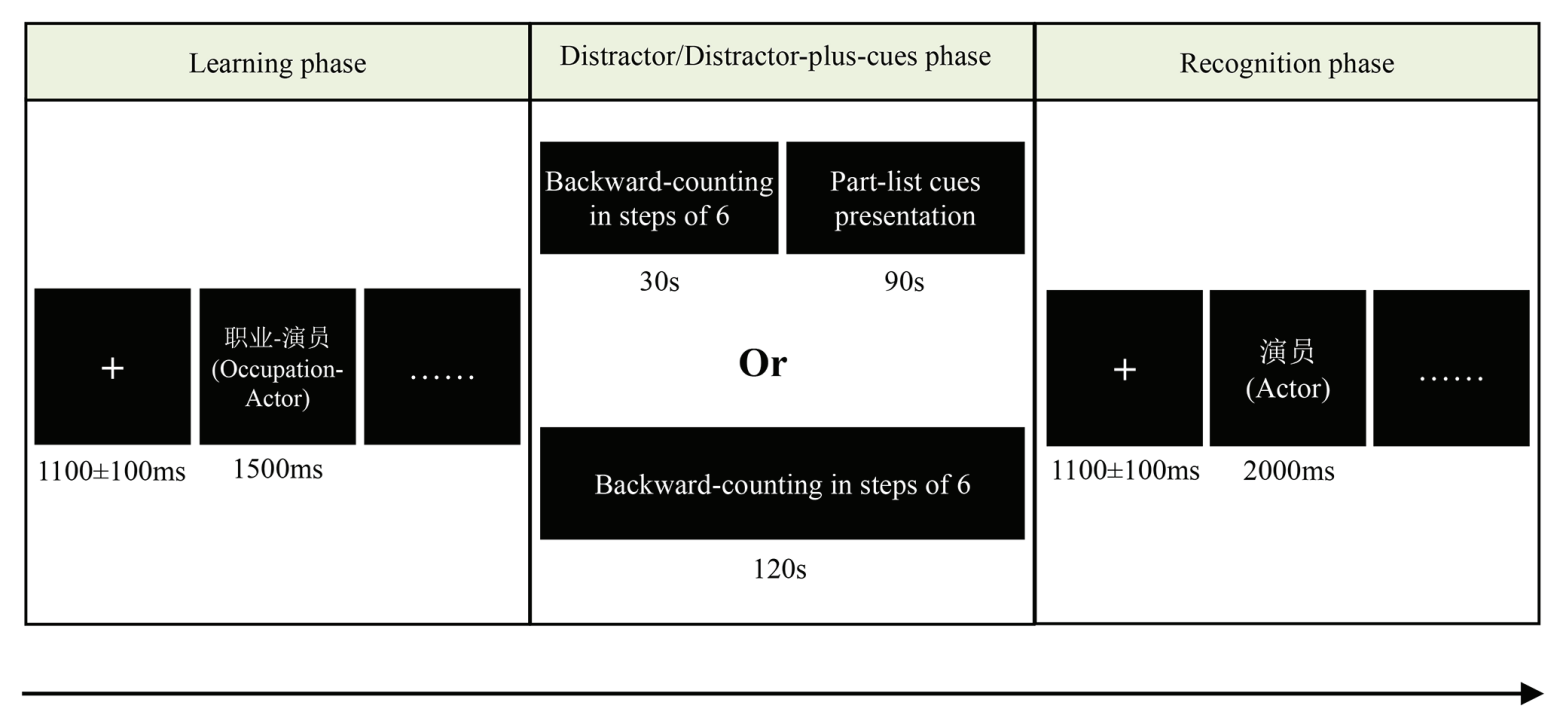
Schematic of trial procedure in learning, distractor/distractor-plus-cues, and recognition phases. English translations of the originally Chinese words are in parentheses.

The experiment included six study-test blocks, each of which consisted of three phases: the learning phase, the distractor/distractor-plus-cues phase, and the recognition phase. The sequence of the six blocks was counterbalanced across participants with restriction of no more than two consecutive blocks belonging to the same cue conditions. The procedure can be seen in Figure 1.

Learning phase: The learning phase included 90 (9 × 10) category-exemplar pairs to study. Before presentation of each category-exemplar, a fixation cross (+) in white font presented in the center of a black screen for 1,000–1,200 ms, and then the category-exemplar in white font presented on the center of the black screen for 1,500 ms. The presentation order of the category-exemplar pairs was randomized within 10 blocks, with each block consisting of one exemplar from each of the nine categories, which resulted in no consecutive category-exemplar pair belonging to the same category.

Distractor/distractor-plus-cues phase: Firstly, the participants were asked to do a distraction task by counting backwards in steps of six on a three-digit number for either 120 s or 30 s (in the no-part-list cue or part-list cue condition, respectively). Then, under the part-list cue condition, 36 (9 × 4) of the 90 studied words were provided as cued items in a pseudorandom order for 90 s. Participants were told to carefully read these items aloud and use these items as retrieval cues to mentally recall the remaining items.

Recognition phase: 81 exemplars (54 studied items, 27 new items, items used as part-list cues did not appear in the recognition phase) appeared successively. Each exemplar was preceded by a presentation of a fixation cross (+) ranging from 1,000 to 1,200 ms. The presentation order of these exemplars was also blocked randomized with restriction of no more than three consecutive exemplars sharing the same correct responses. A Remember/Know/New paradigm following the recommendations by Everaert and Koster (2015) was adopted. Participants were informed about the meaning of Remember/Know/New judgments to find out how to make the corresponding response. A Remember response was to be given if the participants were sure to have seen the item during the learning phase and could recall any specific information associated with that item. A Know response meant that the participants were sure to have seen the item but could not remember any such details. Otherwise, a New response was made to indicate not having seen the item during the learning phase. The above three types of judgments corresponded to three response keys on the keyboard, and the subjects were instructed to use three fingers to press the “Remember,” “Know,” and “New” keys, respectively. Each exemplar presented for 2000 ms, and participants were asked to respond within this period of time.

### Electroencephalogram Recording and Analysis

The EEG data were obtained from 64 channel Ag/AgCl electrodes mounted on an electrode cap (NeuroScan Inc., United States) according to the extended 10-20 system. All recordings were referenced to the left mastoid and re-referenced offline to the average of the left and right mastoid recordings. The electrooculogram (EOG) was recorded bipolarly through electrodes placed above and below the left eye (vertical) and at the outer canthi (horizontal). The impedances was kept below 5 kΩ. The EEG was filtered with a band pass of 0.05–100 Hz and continuously digitized at a sampling rate of 500 Hz. Ocular artifacts were corrected with a regression-based eye movement correction algorithm (Semlitsch et al., 1986) and trials containing eye movement, blinks, excessive muscle activity, or signal drifts were eliminated based on visual inspection. The EEG data were low-pass filtered below 30 Hz (24 dB/octave). All trials with EEG voltages exceeding a threshold of ±75 μV during the recording epoch were excluded from averaging.

Epochs were extracted from the continuous data, lasting from 100 ms before until 1,000 ms after the presentation of each item in the recognition phase, with the −100–0 ms served as baseline correction. ERPs were constructed by averaging EEG data for “Remember” trials (correctly recognized old items with a “Remember” response), “Know” trials (correctly recognized old items with a “Know” response), and “New” trials (correctly rejected new items with a “New” response) for no-part-list cue and part-list cue conditions, resulting in the following six item-type conditions: Remember-No-part-list cue, Remember-Part-list cue, Know-No-part-list cue, Know-Part-list cue, New-No-part-list cue, and New-Part-list cue (average number of artifact-free trials for each condition: 89, 86, 30, 26, 52, and 53, respectively). We can then get the neuro-features of distinctions about recollection and familiarity.

By visually inspecting the grand average ERP waveforms and referring to previous related studies (Friedman et al., 2010; Schaefer et al., 2011; Mollison and Curran, 2012; Hou et al., 2013), the current study extracted mean ERP amplitudes from two time windows (300–500 ms and 500–700 ms) to assess the FN400 and LPC old/new effect, respectively. For each old/new effect, the mean amplitudes of six electrodes taken from frontal and parietal sites (anterior sites: F3, Fz, and F4; posterior sites: P3, Pz, and P4) were measured and analyzed. Neural activity for familiarity was examined by contrasting Know hits with correct rejections, while neural activity for recollection was examined by contrasting Remember hits with Know hits (Li et al., 2016). For each time window, a repeated-measures analysis of variance (ANOVA), which involved cue conditions (part-list cue and no-part-list cue), item types (Remember, Know, and New), and electrodes (F3, Fz, F4, P3, Pz, and P4), was conducted. The Greenhouse-Geisser corrected value of *p* and the Bonferroni-corrected *post-hoc* multiple comparisons were reported in the ANOVA.

## Results

### Behavioral Results

The memory performance for each cue condition in the recognition test is depicted in Tables 1 and 2.

**Table 1:** Mean proportions of Remember and Know responses assigned to hits and false alarms on recognition task, recollection (R) and familiarity (F) scores, and memory discrimination scores (*P_r_*, *d’*) and response bias (*B_r_*) for Remember and Know responses in part-list cue and no-part-list cue conditions.

Condition	Hits		False alarms		Recollection	Familiarity	*P_r(R)_*	*P_r(K)_*	*d’_(R)_*	*d’_(K)_*	*B_r(R)_*	*B_r(K)_*
	Remember response	Know response	Remember response	Know response								
Part-list cue	0.52 (0.03)	0.16 (0.02)	0.11 (0.02)	0.19 (0.03)	0.41 (0.03)	0.13 (0.05)	0.41 (0.03)	−0.03 (0.04)	1.34 (0.13)	−0.07 (0.14)	0.19 (0.03)	0.18 (0.02)
No-part-list cue	0.53 (0.05)	0.20 (0.03)	0.12 (0.02)	0.17 (0.03)	0.41 (0.04)	0.23 (0.05)	0.41 (0.04)	0.03 (0.05)	1.29 (0.10)	0.13 (0.14)	0.24 (0.04)	0.17 (0.02)

Standard errors are in brackets. *P_r(R)_* = discrimination score of Remember responses = P(Remember responses assigned to hits) − P(Remember responses assigned to false alarms); *P_r(K)_* = discrimination score of Know responses = P(Know responses assigned to hits) − P(Know responses assigned to false alarms); *d’_(R)_* = Z(Remember responses assigned to hits) − Z(Remember responses assigned to false alarms); *d’_(K)_* = Z(Know responses assigned to hits) − Z(Know responses assigned to false alarms); *B_r(R)_* = P(Remember responses assigned to false alarms)/[1 − *P_r(R)_*]; *B_r(k)_* = P(Know responses assigned to false alarms)/[1 − *P_r(R)_*]. For *B_r_*, a value less than 0.5 means a conservative bias and a value greater than 0.5 means a liberal bias (Snodgrass and Corwin, 1988).

**Table 2:** Mean reaction times of Remember and Know responses assigned to old items and of new responses assigned to new items on recognition task.

Condition	Remember responses	Know responses	New responses
Part-list cue	1031 (41)	1348 (45)	1143 (38)
No-part-list cue	1008 (38)	1342 (50)	1155 (34)

Standard errors are in brackets.

Because the present study focused particularly on dissociating the recollection (R) and familiarity (F) process, the following formula: R = Remember hits − Remember false alarms, F = [Know hits/(1 − Remember hits)] − [Know false alarms/(1 − Remember false alarms)], proposed by Yonelinas (2002), was employed to estimate the recollection and familiarity processes, respectively. The familiarity decreased significantly in the part-list cue condition compared to the no-part-list cue condition, *t*(16) = −3.271, *p* < 0.01, Cohen’s *d* = 0.796. The recollection was not significantly different between part-list cue and no-part-list cue conditions, *t*(16) = 0.040, *p* > 0.05 (Table 1).

The memory discrimination score [*P_r_* = P(hits) − P(false alarms), *d’* = Z(hits) − Z(false alarms)] and response bias score [*B*_r_ = P(false alarms)]/[1 − (hits − false alarms)] (Snodgrass and Corwin, 1988) were also calculated for Remember and Know items, respectively. The *P_r_* score for Know items decreased marginally significantly in the part-list cue condition compared to the no-part-list cue condition, *t*(16) = −1.980, *p* = 0.065, Cohen’s *d* = 0.480, whereas the *P_r_* score for Remember items was not significantly different between the part-list cue and no-part-list cue conditions, *t*(16) = 0.040, *p* > 0.05. Similarly, the *d’* score for Know items decreased marginally significantly in the part-list cue condition compared to the no-part-list cue condition, *t*(16) = −2.000, *p* = 0.063, Cohen’s *d* = 0.485, whereas the *d’* score for Remember items was not significantly different between part-list cue and no-part-list cue conditions, *t*(16) = 0.468, *p* > 0.05. A similar analysis for response bias also showed a relatively more conservative bias for Remember items under the part-list cue condition than under the no-part-list cue condition, *t*(16) = −2.268, *p* < 0.05, Cohen’s *d* = 0.592, whereas the *B_r_* score for Know items was not significantly different across part-list cue and no-part-list cue conditions, *t*(16) = 1.310, *p* > 0.05 (Table 1).

For RTs, a repeated-measures ANOVA, which involved cue conditions (part-list cue and no-part-list cue) and item types (Remember, Know, and New), was conducted on RTs of Remember and Know responses to studied items and on those of New responses to new items. Main effect of item types [*F*(2, 32) = 18.190, *p* < 0.001, *η*^2^_p_ = 0.532, *MSE* = 912,831.861] revealed that the RTs were faster for Remember items than for New (*p* = 0.07) and Know items (*p* < 0.001) and that the RTs were faster for New items than for Know items (*p* < 0.001; Table 2).

### ERPs Results

**Figure 2:**
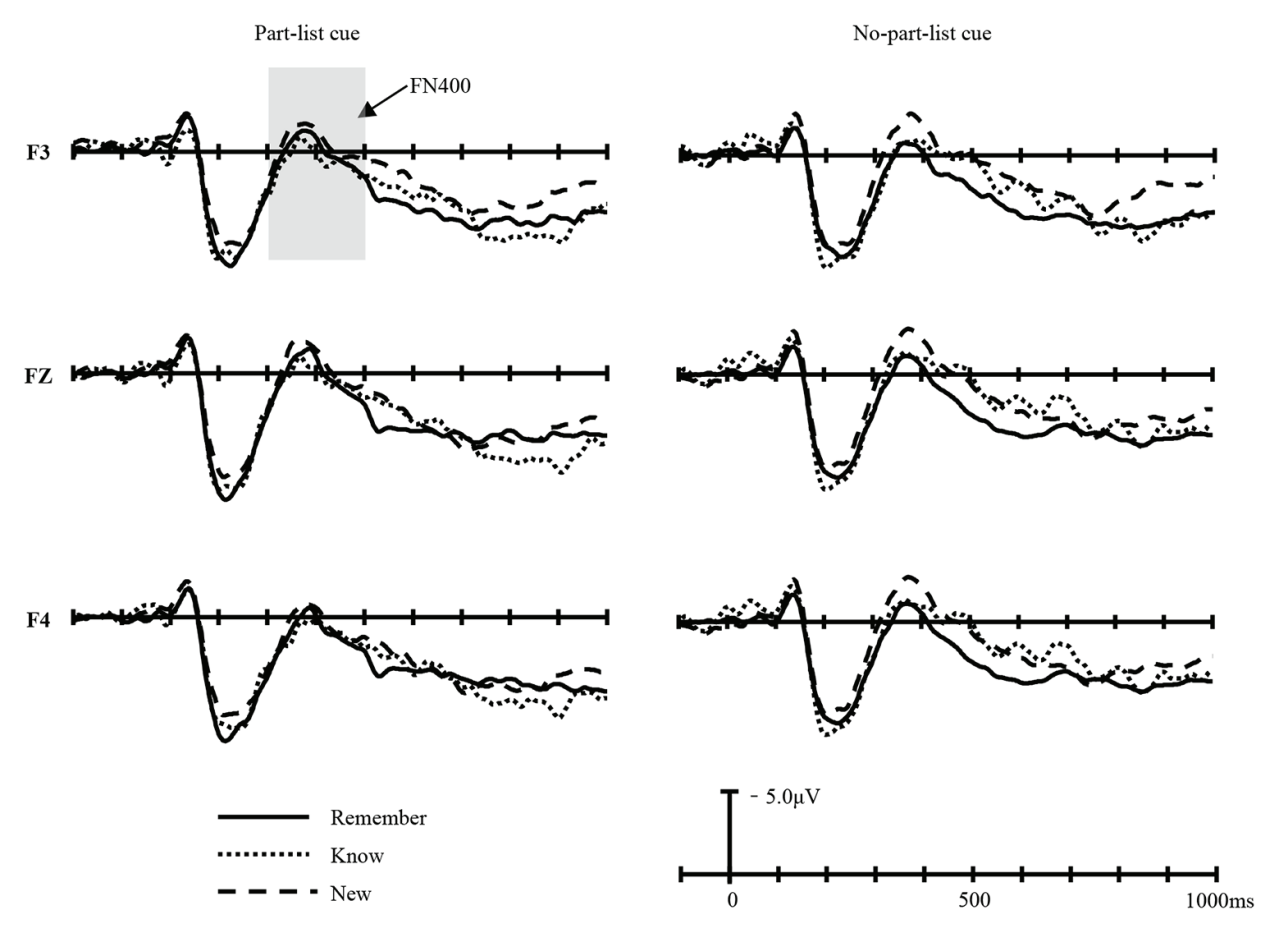
The grand-average waveforms for Remember (Remember responses to old items), Know (Know responses to old items), and New (New responses to new items) items in part-list cue and no-part-list cue conditions at F3, Fz, and F4 electrodes.

The FN400 (300–500 ms), indexing the familiarity process, was measured over electrodes taken from the frontal area. Grand average waveforms for the three item types (Remember, Know, and New) at F3, Fz, and F4 electrodes in the part-list cue and no-part-list cue conditions are shown in Figure 2.

The FN400 amplitudes were entered into a 2 (cue conditions: part-list cue and no-part-list cue) × 3 (item types: Remember, Know, and New) × 6 (electrodes: F3, Fz, F4, P3, Pz, and P4) repeated-measures ANOVA. The main effect of cue conditions was not significant, *F*(1, 16) = 0.045, *p* > 0.05, *η*^2^_p_ = 0.003, *MSE* = 0.550. The main effect of item types was significant, *F*(2, 32) = 3.969, *p* < 0.05, *η*^2^_p_ = 0.199, *MSE* = 44.971. There was also a significant main effect of the electrodes, *F*(5, 80) = 13.741, *p* < 0.05, *η*^2^_p_ = 0.462, *MSE* = 1010.966.The cue conditions × item types effect was significant, *F*(2, 32) = 3.762, *p* < 0.05, *η*^2^_p_ = 0.190, *MSE* = 31.230. The cue conditions × electrodes effect was significant, *F*(5, 80) = 3.429, *p* < 0.05, *η*^2^_p_ = 0.176, *MSE* = 6.909. In addition, the cue conditions × item types × electrodes effect was significant, *F*(10, 160) = 2.829, *p* < 0.05, *η*^2^_p_ = 0.150, *MSE* = 2.922. Simple effect analysis showed that in the part-list cue condition, the differences of mean amplitudes among New, Remember, and Know items were not significant, while in the no-part-list cue condition, the result varies with the region of the electrode sites. For the electrode sites from the frontal region, the mean amplitudes of New items were lower than that of Remember (*p* < 0.01) and that of Know items (*p* < 0.05), and the difference between Remember and Know items was not significant. For the electrode sites from the parietal region, the mean amplitudes of New items were lower than that of Remember (*p* < 0.01) and that of Know items (*p* < 0.05), and no significant difference between other item types was observed (*p*s > 0.05).

**Figure 3:**
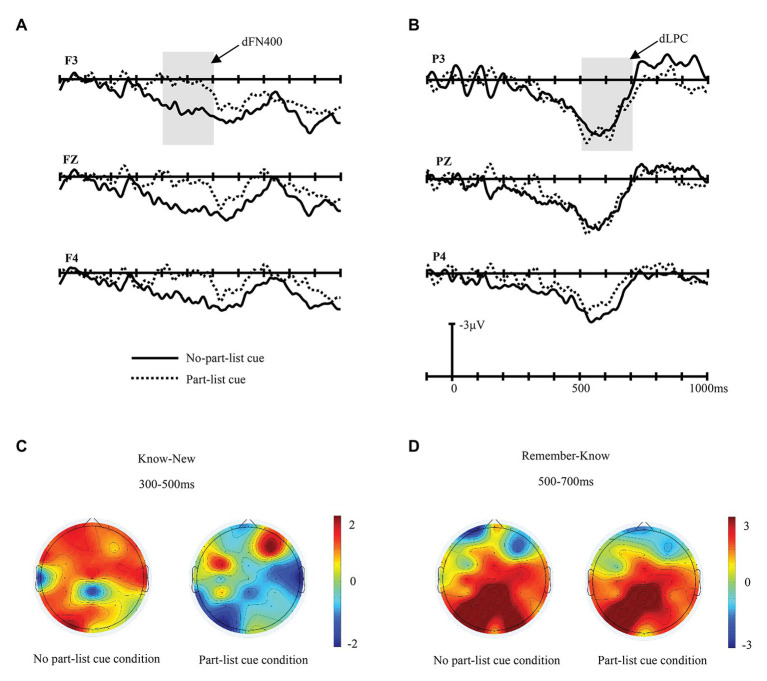
Difference wave of FN400 and late positive complex (LPC). **(A)** dFN400 (ERPs of Know response to old items minus ERPs of New response to new items) at F3, Fz, and F4 electrodes in part-list cue and no-part-list cue conditions, respectively. **(B)** dLPC (ERPs of Remember response to old items minus ERPs of New response to new items) at P3, Pz, and P4 electrodes in part-list cue and no-part-list cue conditions, respectively. **(C)** Topographic maps for dFN400 in the time window of 300–500 ms. **(D)** Topographic maps for dLPC in the time window of 500–700 ms.

To further examine the ERPs associated with familiarity, the FN400 difference waves were calculated by subtracting ERP responses to the trials on which the participant gave a New response to new items from those of Know response to old items. The dFN400 amplitudes of frontal sites were entered into a 2 (cue conditions: part-list cue and no-part-list cue) × 3 (electrodes: F3, Fz, and F4) repeated-measures ANOVA (Figures 3A,C). The main effect of cue conditions was significant, *F*(1, 16) = 7.829, *p* < 0.05, *η*^2^_p_ = 0.329, *MSE* = 54.915, indicating that the amplitudes of dFN400 in the no-part-list cue condition were larger than that of the part-list cue condition. The main effect of electrodes was not significant, *F*(2, 32) = 0.155, *p* > 0.05, *η*^2^_p_ = 0.010, *MSE* = 0.213. In addition, the interactions of electrodes × cue conditions effect was not significant, *F*(2, 32) = 0.050, *p* > 0.05, *η*^2^_p_ = 0.003, *MSE* = 0.197. Also, a 2 (cue conditions: part-list cue and no-part-list cue) × 3 (electrodes: F3, Fz, and F4) repeated-measures ANOVA for the dFN400 amplitudes of parietal sites showed no significant main effects or interactions of cue conditions and electrodes, indicating that the dFN400 is not significantly different under the two cue conditions.

**Figure 4:**
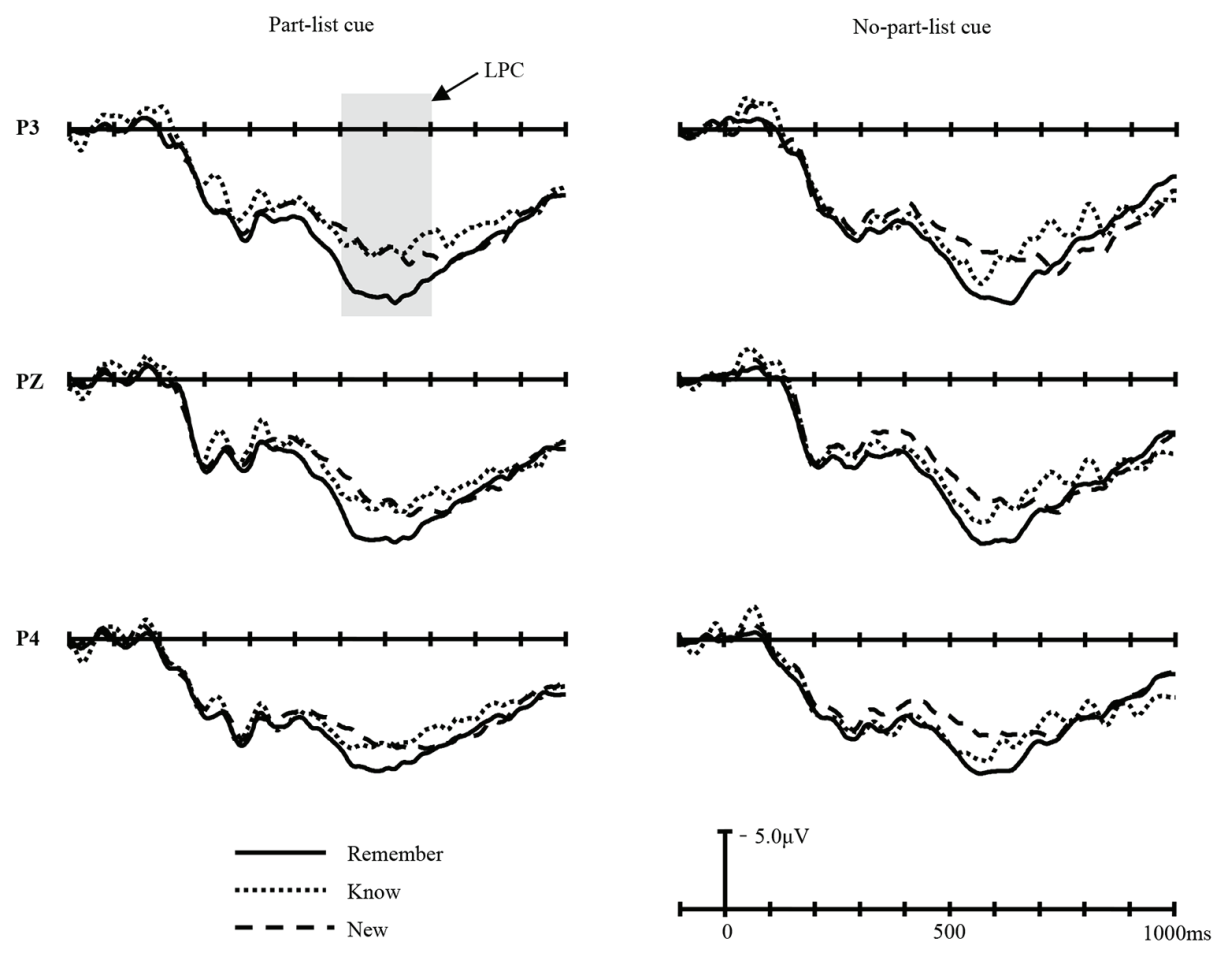
The grand-average waveforms for Remember (Remember responses to old items), Know (Know responses to old items), and New (New responses to new items) items in part-list cue and no-part-list cue conditions at P3, Pz, and P4 electrodes.

The LPC (500–700 ms), indexing the recollection process, was measured over electrodes taken from the parietal area. Grand average waveforms for the three item types (Remember, Know, and New) at P3, P4, and Pz electrodes in the part-list cue and no-part-list cue conditions are shown in Figure 4.

The LPC amplitudes were entered into a 2 (cue conditions: part-list cue and no-part-list cue) × 3 (item types: Remember, Know, and New) × 6 (electrodes: F3, Fz, F4, P3, Pz, and P4) repeated-measures ANOVA. The main effect of cue conditions was not significant, *F*(1, 16) = 0.317, *p* > 0.05, *η*^2^_p_ = 0.019, *MSE* = 4.210. The main effect of item types was significant, *F*(2, 32) = 9.925, *p* < 0.01, *η*^2^_p_ = 0.383, *MSE* = 183.096. There was also a significant main effect of the electrodes, *F*(5, 80) = 12.664, *p* < 0.05, *η*^2^_p_ = 0.442, *MSE* = 996.473. In addition, the interactions between item types and electrodes was significant, *F*(10, 160) = 3.350, *p* < 0.05, *η*^2^_p_ = 0.173, *MSE* = 4.734, revealing a larger amplitude in parietal sites for Remember, Know, and New items (*p*s < 0.05) than in frontal sites and reflecting a significant Remember–Know difference (*p*s < 0.05) and Remember–New difference (*p*s < 0.001) in Pz, P4, P3, and Fz sites.

To further examine the ERPs associated with recollection, the LPC difference waves were calculated by subtracting ERP responses to the trials on which the participant gave a Know response to old items from those of Remember response to old items. The dLPC amplitudes of parietal sites were entered into a 2 (cue conditions: part-list cue and no-part-list cue) × 3 (electrodes: P3, Pz, and P4) repeated-measures ANOVA (Figures 3B,D). The main effect of cue conditions was not significant, *F*(1, 16) = 0.503, *p* > 0.05, *η*^2^_p_ = 0.030, *MSE* = 10.506. The main effect of electrodes was significant, *F*(2, 32) = 5.787, *p* < 0.01, *η*^2^_p_ = 0.266, *MSE* = 10.684. The electrodes × cue conditions effect was not significant, *F*(2, 32) = 0.446, *p* > 0.05, *η*^2^_p_ = 0.027, *MSE* = 0.861. Also, a 2 (cue conditions: part-list cue and no-part-list cue) × 3 (electrodes: F3, Fz, and F4) repeated-measures ANOVA for the dLPC amplitudes of frontal sites showed no significant main effects or interactions of cue conditions and electrodes.

## Discussion

The current study aimed to assess the relative contribution of recollection and familiarity processes to the part-list cuing effect in recognition memory. To achieve this goal, behavioral and ERPs data relevant to the Remember/Know paradigm were recorded and analyzed.

Previous studies focusing on part-list cuing effect scarcely investigated such effect in recognition memory. To date, only a few studies demonstrated part-list cuing effect in a yes/no recognition task (Slamecka, 1975; Todres and Watkins, 1981; Oswald et al., 2006); however, the contribution of recollection and familiarity to recognition memory in these previous studies has not been examined. In line with the previous study (Todres and Watkins, 1981; Oswald et al., 2006), the behavior data of the current study revealed a lowered memory performance under the part-list cue condition than under the part-list cue condition in the recognition test. The further dual-process analysis of the Remember/Know data suggested that the part-list cues lowered the non-cue items’ familiarity but had no significant detrimental effect on the non-cue items’ recollection. Moreover, the analysis of discrimination index *P_r_* and *d’*, which indexes the ability to discriminate between the old and new items, revealed a significant decline for *P_r(K)_* and *d’_(K)_* scores but not the *P_r(R)_* and *d’_(R)_* scores in the part-list cue condition, indicating that part-list cues mainly reduced the ability to discriminate between the old and new items based on the familiarity process. In addition, participants adopted a relatively conservative response bias in both part-list cue and no-part-list cue conditions, but with a relatively more conservative bias for Remember items under the part-list cue condition than under the no-part-list cue condition. These analyses suggested that the participants were better able to discriminate studied from non-studied exemplars in the no-part-list cue condition. In brief, the behavioral results suggested that the part-list cues mainly reduced the familiarity process of the target items, while leaving the recollection process unaffected.

In light of the dual-process theory, recognition retrieval can be divided into two different processes: familiarity and recollection. FN400 in the frontal area reflects familiarity, while LPC in the parietal area reflects recollection (Curran, 2000, 2004; Rugg and Curran, 2007; Schaefer et al., 2011; Ozubko and Yonelinas, 2012; Pergola and Suchan, 2013; Kamp et al., 2016; Bader and Mecklinger, 2017; Küper and Zimmer, 2018; Mecklinger and Bader, 2020). The FN400 old/new effect, which is used to index the familiarity process, is similar in timing and morphology to N400, a correlate related to conceptual priming (Voss and Paller, 2009; Voss et al., 2010, 2012; Kutas and Federmeier, 2011; Pergola et al., 2014). However, previous studies have shown that these two ERP components can be distinguished based on their topography: FN400 has a frontal maximum in the 300–500 ms time window, while N400 has a centro-parietal maximum in the 300–500 ms time window (Bridger et al., 2012; Bader and Mecklinger, 2017; Küper and Zimmer, 2018; Mecklinger and Bader, 2020). The present study found that in the 300–500 ms time window, the difference in amplitude between the old and new items was significantly greater in the frontal region than in the parietal region, which is consistent with the distribution characteristics of FN400, indicating that the old/new effect of 300–500 ms in this study reflects the familiarity process. The present results revealed that in the no-part-list cue condition, the FN400 component is maximal over the frontal area between 300 and 500 ms post-stimulus and the LPC component is maximal over the parietal area between 500 and 700 ms post-stimulus, which is in line with many previous studies mentioned earlier. However, under the part-list cue condition, we did not find the FN400 effect, but the LPC effect. Further analysis revealed that the part-list cuing effect was mostly caused by familiarity change; that is, the reason why part-list cues can lower the recognition performance is that the availability of the stimulus’ familiarity is reduced. The ERP results turned out that the forgetting effect induced by part-list cues was accompanied by a decreased familiarity of the target items, but no significant changes were found in the recollection process, which suggested that part-list cues mainly impaired the target items’ familiarity process, while left the recollection process unaffected in the recognition task.

Although it is widely believed that FN400 in the recognition memory test reflects the familiarity process (Rugg and Curran, 2007), and many studies have assumed that as long as FN400 was observed, it can be inferred that familiarity has occurred. However, many other studies have pointed out that familiarity can occur when adopting conceptually rich stimuli as experimental materials, but conceptual priming often occurs simultaneously with familiarity (Voss and Paller, 2009; Voss et al., 2010, 2012). The learning materials adopted in the present study were category exemplars, which are conceptually rich stimuli. Therefore, although the behavioral performance indicated the occurrence of familiarity, the occurrence of conceptual priming cannot be ruled out. In the present study, when participants indicated familiarity experiences in the recognition test, we believed that these conceptually rich category exemplars would participate in familiarity neural signals plus conceptual priming neural signals, that is, conceptual priming also affects the process of item recognition. Conceptual priming refers to a kind of implicit memory that occurs with the repetition of concept processing. That is, compared to novel conceptual processing, people tend to respond more quickly or accurately to recently repeated processing (Hou et al., 2013). Given that recognition test usually involves repetitive display of meaningful stimuli (first displayed during the encoding phase and then reappeared during the recognition phase; Voss et al., 2010), in the recognition test, the conceptual priming may co-occur with familiarity. Therefore, in addition to the decrease in familiarity, the part-list cuing effect that occurred in the present study may also be accompanied by a decrease in conceptual priming.

The converging behavioral and ERP results of the present study indicated that part-list cue-induced forgetting was accompanied by a reliable decrease in familiarity. However, forgetting was not accompanied by a significant decline in the recollection process. These results are different from those of previous studies that used item-specific probe test and free recall tasks (Aslan et al., 2007; Aslan and Bäuml, 2007; Bäuml and Samenieh, 2012; John and Aslan, 2020). The difference in the role of recollection process in part-list cuing effect obtained in the present study and previous studies may be due to the methodological differences across experiments. When adopting free recall task or item-specific probe test, the number of items that the participants need to learn is relatively small, which enables a better memorization of the items. In the present study, however, the participants were asked to learn 90 exemplars in each study-test block, thus leading to a not-so-good memory of the learned items, which in turn may make the participants rely more on the familiarity process to make judgments in the subsequent memory test.

In prior studies adopting free recall task or item-specific probe test, part-list cuing effect is regarded as the result of retrieval inhibition. According to the retrieval inhibition hypothesis (Anderson et al., 1994; Bäuml and Aslan, 2004, 2006; Aslan et al., 2007), the presence of part-list cues during the recognition phase leads to the implicit retrieval of these items, and this implicit retrieval reduces the general memory representation strength of target items even if the cues were removed during the test. Consistent with the previous studies (Bäuml and Aslan, 2004; Oswald et al., 2006), the present study found that part-list cues impaired the access to non-cues even when these cues were not presented during recognition phase; i.e., the detrimental effect of part-list cues remains largely unaffected when the cues were removed before test. In addition, Bäuml and Aslan (2004) emphasized the importance of controlling the output order to determine the cause of the part-list cuing effect. In order to investigate the above question, they controlled the output order of the test items after the presentation of part-list cues and found a decrease in memory performance, which they thought could be explained by the retrieval inhibition hypothesis. The retrieval inhibition hypothesis assumes that the part-list cuing effect is caused by the decrease in the strength of memory representation of non-cue items (Bäuml and Aslan, 2004; Aslan et al., 2007; John and Aslan, 2020). Therefore, forgetting should be observed regardless of whether the output order is controlled (Aslan and Bäuml, 2007; Bäuml and Samenieh, 2012). In our experiment, the recognition task was used in which the output order was also controlled, and recognition performance was used to measure the target items’ access. For one thing, the lowered recognition accuracy of the target item under the part-list cue condition in the current study replicated the finding of previous studies and also supported the prediction of the retrieval inhibition hypothesis on target items in the recognition task. For another, the results of the present study goes beyond the results of previous studies. Both the behavior and ERP results showed that the reduction in the representation strength of the target items induced by part-list cues is mainly reflected in the decrease of target items’ familiarity. Therefore, we believe that our results can also be explained by the retrieval inhibition hypothesis.

The retrieval inhibition hypothesis believes that the part-list cuing effect has similar inhibitory processes to the retrieval-induced forgetting process. Researchers directly compared the part-list cuing effect with the retrieval-induced forgetting process and found that there was no difference between the two kinds of forgetting whether in quality or in quantity (Bäuml and Kuhbandner, 2003; Bäuml and Aslan, 2004; Zellner and Bäuml, 2005; Aslan and Bäuml, 2009). The results of the present study indicated that part-list cues damage not the recollection but the familiarity process of target items, which corresponds to the results of retrieval-induced forgetting studies (Spitzer and Bäuml, 2007; Rupprecht and Bäuml, 2016). Using the R/K procedure and ROC procedure, Spitzer and Bäuml (2007) investigated the influence of retrieval practice on recognition memory, and they found that retrieval practice mainly reduces the unpracticed items’ familiarity but keeps their recollection largely unaffected, thus supporting the retrieval inhibition hypothesis. Rupprecht and Bäuml (2016) also investigated the retrieval-induced forgetting with the ROC procedure. Although their experiments were not designed to dissociate the recollection and familiarity process of recognition memory, their results illustrated that only inhibition may induce retrieval-induced forgetting in item recognition. Combined with the results of previous studies, we believe that our recognition data are most consistent with the retrieval inhibition hypothesis of the part-list cuing effect.

According to the strategy disruption hypothesis, the forgetting effect caused by part-list cues should be transient, and when removing these cues, the corresponding effect should be eliminated. Since the strategy disruption hypothesis attributes forgetting to changes in the retrieval strategy when cues are provided, removing these cues should enable subjects to use their original retrieval strategies, thereby eliminating any forgetting originally produced by the cues (Basden and Basden, 1995). From the viewpoint of strategy disruption theory, it can be inferred that neither familiarity nor recollection should decrease if part-list cues were not present during the testing phase. The part-list cues in the present study were presented for 90 s in the distractor-plus-cues phase and then disappeared in the following test phase, resulting in no reference being made to using the cues during test, but there was still a significant decrease in familiarity under part-list cue condition, suggesting the existence of impairment effect of part-list cues. The strategy disruption hypothesis holds that when participants are forced to use random retrieval strategies – as the present experiment does when using experimenter-guided presentation order of test items – their original strategies would be destroyed regardless of whether part-list cues are provided (Peynircioğlu, 1989). The finding that reliable harmful effects of part-list cues exist in recognition memory thus contradicts explanations solely based on strategy disruption. In sum, strategy disruption predicts no part-list cuing impairment in the recognition task, which was not proven by the results of the present study.

In sum, this study showed that the lowered performance caused by the presentation of part-list cues in item recognition is mainly attributed to the reduction of familiarity but not recollection. This finding of no reliable impairment of recollection process corresponds to previous studies focusing on retrieval-induced forgetting (Spitzer and Bäuml, 2007; Rupprecht and Bäuml, 2016). Moreover, the results of the present study can also provide experimental evidence for the retrieval inhibition hypothesis.

## Data Availability Statement

The raw data supporting the conclusions of this article will be made available by the authors, without undue reservation.

## Ethics Statement

The studies involving human participants were reviewed and approved by Human Research Ethics Committee of Xinyang Normal University. The patients/participants provided their written informed consent to participate in this study.

## Author Contributions

TL and XB designed the experiment. MX collected the data. TL analyzed the data. TL and MX wrote the manuscript. TL, MX, and XB revised the manuscript. All authors contributed to the article and approved the submitted version.

### Conflict of Interest

The authors declare that the research was conducted in the absence of any commercial or financial relationships that could be construed as a potential conflict of interest.

## References

[ref1] Anderson M. C. Bjork R. A. Bjork E. L. (1994). Remembering can cause forgetting: retrieval dynamics in long-term memory. J. Exp. Psychol. Learn. Mem. Cogn. 20, 1063–1087. 10.1037/0278-7393.20.5.1063 7931095

[ref2] Andrés P. (2009). Equivalent part set cueing effects in younger and older adults. Eur. J. Cogn. Psychol. 21, 176–191. 10.1080/09541440802033980

[ref3] Andrés P. Howard C. E. (2011). Part set cuing in older adults: further evidence of intact forgetting in aging. Neuropsychol. Dev. Cogn. B Aging Neuropsychol. Cogn. 18, 385–395. 10.1080/13825585.2010.542892, PMID: 21728887

[ref4] Aslan A. Bäuml K. -H. (2007). Part-list cuing with and without item-specific probes: the role of encoding. Psychon. Bull. Rev. 14, 489–494. 10.3758/bf03194095, PMID: 17874594

[ref5] Aslan A. Bäuml K. -H. (2009). The role of item similarity in part-list cueing impairment. Memory 17, 697–707. 10.1080/09658210902740886, PMID: 19575327

[ref6] Aslan A. Bäuml K. -H. Grundgeiger T. (2007). The role of inhibitory processes in part-list cuing. J. Exp. Psychol. Learn. Mem. Cogn. 33, 335–341. 10.1037/0278-7393.33.2.335, PMID: 17352615

[ref7] Aslan A. John T. (2019). Part-list cuing effects in younger and older adults’ episodic recall. Psychol. Aging 34, 262–267. 10.1037/pag0000268, PMID: 29927271

[ref8] Baddeley A. Eysenck M. W. Anderson M. C. (2014). Memory. 2nd Edn. Hoboken: Taylor and Francis.

[ref9] Bader R. Mecklinger A. (2017). Separating event-related potential effects for conceptual fluency and episodic familiarity. J. Cogn. Neurosci. 29, 1402–1414. 10.1162/jocn_a_01131, PMID: 28387586

[ref10] Barber S. J. Harris C. B. Rajaram S. (2015). Why two heads apart are better than two heads together: multiple mechanisms underlie the collaborative inhibition effect in memory. J. Exp. Psychol. Learn. Mem. Cogn. 41, 559–566. 10.1037/xlm0000037, PMID: 25068855 PMC4309738

[ref11] Barker G. R. Warburton E. C. (2020). Multi-level analyses of associative recognition memory: the whole is greater than the sum of its parts. Curr. Opin. Behav. Sci. 32, 80–87. 10.1016/j.cobeha.2020.02.004, PMID: 32617383 PMC7323598

[ref12] Basden D. R. Basden B. H. (1995). Some tests of the strategy disruption interpretation of part-list cuing inhibition. J. Exp. Psychol. Learn. Mem. Cogn. 21, 1656–1669. 10.1037/0278-7393.21.6.1656

[ref13] Basden D. R. Basden B. H. Galloway B. C. (1977). Inhibition with part-list cuing: some tests of the item strength hypothesis. J. Exp. Psychol. Hum. Learn. Mem. 3, 100–108. 10.1037/0278-7393.3.1.100

[ref14] Basden B. H. Basden D. R. Stephens S. P. (2002). Part-set cuing of order information in recall tests. J. Mem. Lang. 47, 517–529. 10.1016/s0749-596x(02)00016-5

[ref15] Bastin C. Besson G. Simon J. Delhaye E. Geurten M. Willems S.. (2019). An integrative memory model of recollection and familiarity to understand memory deficits. Behav. Brain Sci. 42:e281. 10.1017/S0140525X19000621, PMID: 30719958

[ref16] Bäuml K. -H. Aslan A. (2004). Part-list cuing as instructed retrieval inhibition. Mem. Cogn. 32, 610–617. 10.3758/BF03195852, PMID: 15478755

[ref17] Bäuml K. -H. Aslan A. (2006). Part-list cuing can be transient and lasting: the role of encoding. J. Exp. Psychol. Learn. Mem. Cogn. 32, 33–43. 10.1037/0278-7393.32.1.33, PMID: 16478338

[ref18] Bäuml K. -H. Kissler J. Rak A. (2002). Part-list cuing in amnesic patients: evidence for a retrieval deficit. Mem. Cogn. 30, 862–870. 10.3758/bf03195772, PMID: 12450090

[ref19] Bäuml K. -H. Kuhbandner C. (2003). Retrieval-induced forgetting and part-list cuing in associatively structured lists. Mem. Cogn. 31, 1188–1197. 10.3758/bf03195802, PMID: 15058680

[ref20] Bäuml K. -H. Samenieh A. (2012). Influences of part-list cuing on different forms of episodic forgetting. J. Exp. Psychol. Learn. Mem. Cogn. 38, 366–375. 10.1037/a0025367, PMID: 21928931

[ref21] Bierstaker J. L. (2003). Auditor recall and evaluation of internal control information: does task-specific knowledge mitigate part-list interference? Manag. Audit. J. 18, 90–99. 10.1108/02686900310455074

[ref22] Bovee J. C. Fitz C. Yehl G. Parrott S. Kelley M. R. (2009). “Applied part-set cuing” in Applied memory. ed. Kelley M. (Hauppauge, NY: Nova Science), 73–87.

[ref23] Bridger E. K. Bader R. Kriukova O. Unger K. Mecklinger A. (2012). The FN400 is functionally distinct from the N400. NeuroImage 63, 1334–1342. 10.1016/j.neuroimage.2012.07.047, PMID: 22850570

[ref24] Brown J. (1968). Reciprocal facilitation and impairment of free recall. Psychon. Sci. 10, 41–42. 10.3758/bf03331397

[ref25] Christensen B. K. Girard T. A. Benjamin A. S. Vidailhet P. (2006). Evidence for impaired mnemonic strategy use among patients with schizophrenia using the part-list cuing paradigm. Schizophr. Res. 85, 1–11. 10.1016/j.schres.2006.03.001, PMID: 16632330

[ref26] Costanzo F. Vicari S. Carlesimo G. A. (2013). Familiarity and recollection in Williams syndrome. Cortex 49, 232–242. 10.1016/j.cortex.2011.06.007, PMID: 21774924

[ref27] Crescentini C. Shallice T. Del Missier F. Macaluso E. (2010). Neural correlates of episodic retrieval: an fMRI study of the part-list cueing effect. NeuroImage 50, 678–692. 10.1016/j.neuroimage.2009.12.114, PMID: 20060480

[ref28] Curran T. (2000). Brain potentials of recollection and familiarity. Mem. Cogn. 28, 923–938. 10.3758/BF03209340, PMID: 11105518

[ref29] Curran T. (2004). Effects of attention and confidence on the hypothesized ERP correlates of recollection and familiarity. Neuropsychologia 42, 1088–1106. 10.1016/j.neuropsychologia.2003.12.011, PMID: 15093148

[ref30] Dew I. T. Cabeza R. (2013). A broader view of perirhinal function: from recognition memory to fluency-based decisions. J. Neurosci. 33, 14466–14474. 10.1523/jneurosci.1413-13.2013, PMID: 24005298 PMC3761052

[ref31] Evans L. H. Wilding E. L. (2012). Recollection and familiarity make independent contributions to memory judgments. J. Neurosci. 32, 7253–7257. 10.1523/JNEUROSCI.6396-11.2012, PMID: 22623670 PMC6622307

[ref32] Everaert J. Koster E. H. W. (2015). Interactions among emotional attention, encoding, and retrieval of ambiguous information: an eye-tracking study. Emotion 15, 539–543. 10.1037/emo0000063, PMID: 25775233

[ref33] Friedman D. de Chastelaine M. Nessler D. Malcolm B. (2010). Changes in familiarity and recollection across the lifespan: an ERP perspective. Brain Res. 1310, 124–141. 10.1016/j.brainres.2009.11.016, PMID: 19914220 PMC2812671

[ref34] Gao H. Qi M. Zhang Q. (2019). Elaborately rehearsed information can be forgotten: a new paradigm to investigate directed forgetting. Neurobiol. Learn. Mem. 164:107063. 10.1016/j.nlm.2019.107063, PMID: 31376463

[ref35] Horne E. D. Koen J. D. Hauck N. Rugg M. D. (2020). Age differences in the neural correlates of the specificity of recollection: an event-related potential study. Neuropsychologia 140:107394. 10.1016/j.neuropsychologia.2020.107394, PMID: 32061829 PMC7078048

[ref36] Hou M. Gao C. Wu J. Guo C. (2014). Neural correlates of familiarity and conceptual fluency are dissociable at encoding. Chin. Sci. Bull. 59, 3602–3609. 10.1007/s11434-014-0392-5

[ref37] Hou M. Safron A. Paller K. A. Guo C. (2013). Neural correlates of familiarity and conceptual fluency in a recognition test with ancient pictographic characters. Brain Res. 1518, 48–60. 10.1016/j.brainres.2013.04.041, PMID: 23632379

[ref38] John T. Aslan A. (2018). Part-list cuing effects in children: a developmental dissociation between the detrimental and beneficial effect. J. Exp. Child Psychol. 166, 705–712. 10.1016/j.jecp.2017.08.013, PMID: 28943058

[ref39] John T. Aslan A. (2020). Age differences in the persistence of part-list cuing impairment: the role of retrieval inhibition and strategy disruption. J. Exp. Child Psychol. 191:104746. 10.1016/j.jecp.2019.104746, PMID: 31839266

[ref40] Johnson J. D. Suzuki M. Rugg M. D. (2013). Recollection, familiarity, and content-sensitivity in lateral parietal cortex: a high-resolution fMRI study. Front. Hum. Neurosci. 7:219. 10.3389/fnhum.2013.00219, PMID: 23734122 PMC3661949

[ref41] Kamp S. M. Bader R. Mecklinger A. (2016). The effect of unitizing word pairs on recollection versus familiarity-based retrieval‐ further evidence from ERPs. Adv. Cogn. Psychol. 12, 169–178. 10.5709/acp-0196-2, PMID: 28154613 PMC5279856

[ref42] Kimbal D. R. Bjork E. L. Bjork R. A. Smith T. A. (2008). Part-list cuing and the dynamics of false recall. Psychon. Bull. Rev. 15, 296–301. 10.3758/pbr.15.2.296, PMID: 18488643 PMC2914858

[ref43] King D. R. de Chastelaine M. Elward R. L. Wang T. H. Rugg M. D. (2018). Dissociation between the neural correlates of recollection and familiarity in the striatum and hippocampus: across-study convergence. Behav. Brain Res. 354, 1–7. 10.1016/j.bbr.2017.07.031, PMID: 28803854 PMC5809243

[ref44] Kissler J. Bäuml K. -H. (2005). Memory retrieval in schizophrenia: evidence from part-list cuing. J. Int. Neuropsychol. Soc. 11, 273–280. 10.1017/S1355617705050320, PMID: 15892903

[ref45] Küper K. Zimmer H. D. (2018). The impact of perceptual changes to studied items on ERP correlates of familiarity and recollection is subject to hemispheric asymmetries. Brain Cogn. 122, 17–25. 10.1016/j.bandc.2018.01.006, PMID: 29396208

[ref46] Kutas M. Federmeier K. D. (2011). Thirty years and counting: finding meaning in the N400 component of the event-related brain potential (ERP). Annu. Rev. Psychol. 62, 621–647. 10.1146/annurev.psych.093008.131123, PMID: 20809790 PMC4052444

[ref47] Lehmer E. M. Bäuml K. T. (2018). Part-list cuing can impair, improve, or not influence recall performance: the critical roles of encoding and access to study context at test. J. Exp. Psychol. Learn. Mem. Cogn. 44, 1186–1200. 10.1037/xlm0000517, PMID: 29578736

[ref48] Li B. Mao X. Wang Y. Guo C. (2017). Electrophysiological correlates of familiarity and recollection in associative recognition: contributions of perceptual and conceptual processing to unitization. Front. Hum. Neurosci. 11:125. 10.3389/fnhum.2017.00125, PMID: 28400723 PMC5369601

[ref49] Li B. Wang W. Gao C. Guo C. (2016). Masked repetition priming hinders subsequent recollection but not familiarity: a behavioral and event-related potential study. Cogn. Affect. Behav. Neurosci. 16, 789–801. 10.3758/s13415-016-0431-6, PMID: 27197527

[ref50] Marsh E. J. Dolan P. O. Balota D. A. Roediger H. L. (2004). Part-set cuing effects in younger and older adults. Psychol. Aging 19, 134–144. 10.1037/0882-7974.19.1.134 15065937

[ref51] Mecklinger A. Bader R. (2020). From fluency to recognition decisions: a broader view of familiarity-based remembering. Neuropsychologia 146:107527. 10.1016/j.neuropsychologia.2020.107527, PMID: 32540265

[ref52] Meyer P. Mecklinger A. Friederici A. D. (2010). On the processing of semantic aspects of experience in the anterior medial temporal lobe: an event-related fMRI study. J. Cogn. Neurosci. 22, 590–601. 10.1162/jocn.2009.21199, PMID: 19301996

[ref53] Mickes L. Seale-Carlisle T. M. Wixted J. T. (2013). Rethinking familiarity: Remember/Know judgments in free recall. J. Mem. Lang. 68, 333–349. 10.1016/j.jml.2013.01.001, PMID: 23637470 PMC3637981

[ref54] Mollison M. V. Curran T. (2012). Familiarity in source memory. Neuropsychologia 50, 2546–2565. 10.1016/j.neuropsychologia.2012.06.027, PMID: 22789677 PMC3432179

[ref55] Muntean W. J. Kimball D. R. (2012). Part-set cueing and the generation effect: an evaluation of a two-mechanism account of part-set cueing. J. Cogn. Psychol. 24, 957–964. 10.1080/20445911.2012.720967

[ref56] Oswald K. M. Serra M. Krishna A. (2006). Part-list cuing in speeded recognition and free recall. Mem. Cogn. 34, 518–526. 10.3758/BF03193575, PMID: 16933761

[ref57] Ozubko J. D. Yonelinas A. P. (2012). A familiar finding: pseudowords are more familiar but no less recollectable than words. J. Mem. Lang. 66, 361–375. 10.1016/j.jml.2011.11.002

[ref58] Pei B. K. W. Tuttle B. M. (1999). Part-set cueing effects in a diagnostic setting with professional auditors. J. Behav. Decis. Mak. 12, 233–256. 10.1002/(sici)1099-0771(199909)12:3<233::Aid-bdm326>3.0.Co;2-m

[ref59] Pergola G. Bellebaum C. Suchan B. (2014). First come, last primed: FN400 reflects post-encoding editing of the memory trace. Behav. Brain Res. 266, 63–76. 10.1016/j.bbr.2014.02.050, PMID: 24631391

[ref60] Pergola G. Suchan B. (2013). Associative learning beyond the medial temporal lobe: many actors on the memory stage. Front. Behav. Neurosci. 7:162. 10.3389/fnbeh.2013.00162, PMID: 24312029 PMC3832901

[ref61] Peynircioğlu Z. F. (1989). Part-set cuing effect with word-fragment cuing: evidence against the strategy disruption and increased-list-length explanations. J. Exp. Psychol. Learn. Mem. Cogn. 15, 147–152. 10.1037/0278-7393.15.1.147

[ref62] Radvansky G. A. Tamplin A. K. (2013). Suppression in retrieval practice, part-set cueing, and negative priming memory: the hydrogen model. Q. J. Exp. Psychol. 66, 1368–1398. 10.1080/17470218.2012.743572, PMID: 23170860

[ref63] Reysen M. B. Nairne J. S. (2002). Part-set cuing of false memories. Psychon. Bull. Rev. 9, 389–393. 10.3758/bf03196298, PMID: 12120805

[ref64] Roediger H. L. Stellon C. C. Tulving E. (1977). Inhibition from part-list cues and rate of recall. J. Exp. Psychol. Hum. Learn. Mem. 3, 174–188. 10.1037/0278-7393.3.2.174

[ref65] Rosenstreich E. Goshen-Gottstein Y. (2015). Recollection-based retrieval is influenced by contextual variation at encoding but not at retrieval. PLoS One 10:e0130403. 10.1371/journal.pone.0130403, PMID: 26135583 PMC4489907

[ref66] Rugg M. D. Curran T. (2007). Event-related potentials and recognition memory. Trends Cogn. Sci. 11, 251–257. 10.1016/j.tics.2007.04.004 17481940

[ref67] Rupprecht J. Bäuml K. -H. T. (2016). Retrieval-induced forgetting in item recognition: retrieval specificity revisited. J. Mem. Lang. 86, 97–118. 10.1016/j.jml.2015.09.003

[ref68] Schaefer A. Pottage C. L. Rickart A. J. (2011). Electrophysiological correlates of remembering emotional pictures. NeuroImage 54, 714–724. 10.1016/j.neuroimage.2010.07.030, PMID: 20650320

[ref69] Semlitsch H. V. Anderer P. Schuster P. Presslich O. (1986). A solution for reliable and valid reduction of ocular artifacts, applied to the P300 ERP. Psychophysiology 23, 695–703. 10.1111/j.1469-8986.1986.tb00696.x 3823345

[ref70] Shaffer R. A. Mcdermott K. B. (2020). A role for familiarity in supporting the testing effect over time. Neuropsychologia 138:107298. 10.1016/j.neuropsychologia.2019.107298, PMID: 31838098

[ref71] Slamecka N. J. (1968). An examination of trace storage in free recall. J. Exp. Psychol. 76, 504–513. 10.1037/h0025695 5650563

[ref72] Slamecka N. J. (1975). Intralist cueing of recognition. J. Verbal Learn. Verbal Behav. 14, 630–637. 10.1016/s0022-5371(75)80050-8

[ref73] Sloman S. A. Bower G. H. Rohrer D. (1991). Congruency effects in part-list cuing inhibition. J. Exp. Psychol. Learn. Mem. Cogn. 17, 974–982. 10.1037//0278-7393.17.5.974 1834778

[ref74] Snodgrass J. G. Corwin J. (1988). Pragmatics of measuring recognition memory: applications to dementia and amnesia. J. Exp. Psychol. Gen. 117, 34–50. 10.1037/0096-3445.117.1.34 2966230

[ref75] Spitzer B. Bäuml K. -H. (2007). Retrieval-induced forgetting in item recognition: evidence for a reduction in general memory strength. J. Exp. Psychol. Learn. Mem. Cogn. 33, 863–875. 10.1037/0278-7393.33.5.863, PMID: 17723065

[ref76] Stróżak P. Abedzadeh D. Curran T. (2016a). Separating the FN400 and N400 potentials across recognition memory experiments. Brain Res. 1635, 41–60. 10.1016/j.brainres.2016.01.015, PMID: 26776478 PMC4779423

[ref77] Stróżak P. Bird C. W. Corby K. (2016b). FN400 and LPC memory effects for concrete and abstract words. Psychophysiology 53, 1669–1678. 10.1111/psyp.12730, PMID: 27463978 PMC5061608

[ref78] Todres A. K. Watkins M. J. (1981). A part-set cuing effect in recognition memory. J. Exp. Psychol. Hum. Learn. Mem. 7, 91–99. 10.1037/0278-7393.7.2.91

[ref79] Tousignant C. Bodner G. E. Arnold M. M. (2015). Effects of context on recollection and familiarity experiences are task dependent. Conscious. Cogn. 33, 78–89. 10.1016/j.concog.2014.11.011, PMID: 25543993

[ref80] Tulving E. (1985). Memory and consciousness. Canadian psychology. Psychol. Can. 26, 1–12. 10.1037/h0080017

[ref81] Tulving E. Pearlstone Z. (1966). Availability versus accessibility of information in memory for words. J. Verbal Learn. Verbal Behav. 5, 381–391. 10.1016/S0022-5371(66)80048-8

[ref82] Ventura-Bort C. Wendt J. Wirkner J. König J. Lotze M. Hamm A. O.. (2020). Neural substrates of long-term item and source memory for emotional associates: an fMRI study. Neuropsychologia 147:107561. 10.1016/j.neuropsychologia.2020.107561, PMID: 32712148

[ref83] Voss J. L. Lucas H. D. Paller K. A. (2010). Conceptual priming and familiarity: different expressions of memory during recognition testing with distinct neurophysiological correlates. J. Cogn. Neurosci. 22, 2638–2651. 10.1162/jocn.2009.21341, PMID: 19702474

[ref84] Voss J. L. Lucas H. D. Paller K. A. (2012). More than a feeling: pervasive influences of memory without awareness of retrieval. Cogn. Neurosci. 3, 193–207. 10.1080/17588928.2012.674935, PMID: 24171735 PMC4385384

[ref85] Voss J. L. Paller K. A. (2009). Remembering and knowing: electrophysiological distinctions at encoding but not retrieval. NeuroImage 46, 280–289. 10.1016/j.neuroimage.2009.01.048, PMID: 19457375

[ref86] Voss J. L. Paller K. A. (2017). “Neural substrates of remembering: event-related potential studies” in Learning and memory: A comprehensive reference. ed. J. H. Byrne (Oxford: Academic Press), 81–98.

[ref87] Wang T. H. de Chastelaine M. Minton B. Rugg M. D. (2012). Effects of age on the neural correlates of familiarity as indexed by ERPs. J. Cogn. Neurosci. 24, 1055–1068. 10.1162/jocn_a_00129, PMID: 21878056 PMC3262081

[ref88] Wang W. Li B. Gao C. Xiao X. Guo C. (2015). Electrophysiological correlates associated with contributions of perceptual and conceptual fluency to familiarity. Front. Hum. Neurosci. 9:321. 10.3389/fnhum.2015.00321, PMID: 26097450 PMC4456582

[ref89] Wang Y. Mao X. Li B. Wang W. Guo C. (2016). Dissociating the electrophysiological correlates between item retrieval and associative retrieval in associative recognition: from the perspective of directed forgetting. Front. Psychol. 7:1754. 10.3389/fpsyg.2016.01754, PMID: 27872605 PMC5098155

[ref90] Yonelinas A. P. (2002). The nature of recollection and familiarity: a review of 30 years of research. J. Mem. Lang. 46, 441–517. 10.1006/jmla.2002.2864

[ref91] Yonelinas A. P. Jacoby L. L. (2012). The process-dissociation approach two decades later: convergence, boundary conditions, and new directions. Mem. Cogn. 40, 663–680. 10.3758/s13421-012-0205-5, PMID: 22528824

[ref92] Yoon C. Feinberg F. Luo T. Hedden T. Gutchess A. H. Chen H. Y. M.. (2004). A cross-culturally standardized set of pictures for younger and older adults: American and Chinese norms for name agreement, concept agreement, and familiarity. Behav. Res. Methods Instrum. Comput. 36, 639–649. 10.3758/bf03206545, PMID: 15641410

[ref93] Zellner M. Bäuml K. -H. (2005). Intact retrieval inhibition in children’s episodic recall. Mem. Cogn. 33, 396–404. 10.3758/bf03193058, PMID: 16156176

